# Association between Gastric Pathology and Hepatitis B Virus Infection in Patients with or without *Helicobacter Pylori*

**DOI:** 10.31557/APJCP.2019.20.7.2177

**Published:** 2019

**Authors:** Mahmud Baghbanian, Seyyed Abolfazl Hoseini Mousa, Masoud Doosti, Mansour Moghimi

**Affiliations:** 1 *Department of Internal Medicine, *; 4 *Department of Pathology, School of Medicine, *; 2 *Infectious and Tropical Diseases Research Center, Shahid Sadoughi University of Medical Science, Yazd, *; 3 *Department of Medical Virology, Faculty of Medical Sciences, Tarbiat Modares University (TMU), Tehran, Iran. *

**Keywords:** Gastric cancer- Hepatitis B virus- Helicobacter pylori

## Abstract

**Background:**

In the recent years, hepatitis B virus (HBV) infection has been considered as a risk factor for gastric cancer, but further studies are required to confirm these claim. The present study was aimed to evaluate the correlation between gastric pathology (precancerous and cancerous conditions) with HBV infection in *Helicobacter pylori* (*H. pylori*) positive or negative patients.

**Methods:**

In this cross-sectional study, 728 patients under endoscopy examination in Yazd Shaheed Sadoughi Hospital between 2017 and 2018 were participated. Histopathological analysis was performed on gastric specimens. Hepatitis B surface antigen (HBsAg) in sera was detected by the enzyme-linked immunosorbent assay (ELISA). The relationship between gastric pathology and HBV infection were explored by logistic regression analysis.

**Results:**

Of 728 patients, HBsAg and* H. pylori* infection were detected in 83 and 408 patients, respectively. Sixty nine patients were co-infected with* H. pylori*/HBV.* H. pylori* infection detected in patients with HbsAg positive significantly more than those were negative for HbsAg (p=0.029). None of HBV/H. pylori co-infected patients did not have normal gastric tissue. A significant difference was seen in histopathology of gastric tissue between HBsAg positive patients with and without* H. pylori* infection (p<0.0001). The HBsAg was associated with histopathology of gastric (OR=21.56, 95℅CI=7.070-65.741, p<0.001) and as a risk factor for gastritis (OR=12.457, 95℅CI= 3.007-51.614, P=0.001) but no cancer (OR=2.127, 95℅CI=0.242-18.704, P=0.496).

**Conclusion:**

The HBV infection alone is associated with some precancerous lesions but is not correlated with gastric cancer. It can increase development of premalignant conditions and carcinoma significantly in* H. pylori* positive patients.

## Introduction

The gastric cancer is the fourth most commonly diagnosed cancer in men and fifth in women worldwide. A decrease in the incidence rate of gastric cancer has shown in developed countries during recent decades, but the mortality rate associated with this malignancy is still high (Ge et al., 2018). The incidence rate of this cancer in Iran is 158 cases per 100,000 peoples. A cascade of pathology changes, including non-atrophic gastritis, atrophic gastritis, intestinal metaplasia, and dysplasia happens to finally create stomach carcinoma (Mera et al., 2018). Some infectious agents can affect gastric tissue (Fattahi et al., 2018). *Helicobacter pylori* (*H. pylori*) is the most common risk factor for changing of normal gastric tissue to cancer (Mera et al., 2018). The association of Epstein–Barr virus (EBV) and cytomegalovirus (CMV) infection with stomach cancer is controversial (Fattahi et al., 2018; Varga et al., 2018). Other risk factors include genetic factors, family history, smoking, diabetes and alcohol drinking (Lochhead and El-Omar, 2018). 

**Table 1:** Frequency Distribution of Population in Terms of Variables under Study

Characteristic	n	Percentage
Sex		
Male	370	50.8
Female	358	49.2
Addiction		
Yes	23	3.2
No	705	96.8
HBV infection		
HBsAg positive	83	7.57
HBsAg negative	645	92.43
*H. pylori* infection		
Positive	408	56
Negative	320	44
HBV/*H. pylori* co-infection		
Yes	69	9.4
No	659	90.6
Histopathology of gastric		
Normal	259	35.6
Gastritis	292	40.1
Atrophic gastritis	60	8.2
Metaplasia	63	8.7
Dysplasia	19	2.6
Cancer	35	4.8

**Table 2:** Association between Some Variables under Study with HBsAg Positivity

Variable	HbsAg positive	HbsAg negative	P value
Mean age	53.63±14.40	42.70±12.62	0.043
Sex			
Male	56 (67.5٪)	314 (48.7٪)	0.001
Female	27 (32.5٪)	331 (51.3٪)	
Addiction			
Yes	11 (13.3٪)	12 (1.98٪)	0.031
No	72 (86.7٪)	633 (98.02٪)	
*H. pylori* infection			
Positive	69 (83.1٪)	339 (52.6)	0.029
Negative	14 (16.9٪)	306 (47.4٪)	

**Table 3:** Comparison of Histopathology Findings between HBsAg Positive and HBsAg Negative Patients

HBV infection	*H. pylori* infection	Histopathology of gastric						total	P value
		Normal	gastritis	Atrophic gastritis	Metaplasia	Dysplasia	Cancer		
HBsAg positive	Positive	0	28 (3.8)	14 (1.9)	10 (1.3)	5 (0.6)	8 (1)	69 (9)	<0.0001
	Negative	5 (0.6)	4 (0.5)	6 (0.8)	2 (0.2)	0	1 (0.1)	14 (1.9)	
HBsAg negative	Positive	55 (7.5)	255 (35)	40 (5.4)	42 (5.7)	10 (1.3)	20 (2.7)	339 (46.5)	0.003
	Negative	199 (27.3)	5 (0.6)	0	9 (1.2)	4 (0.5)	6 (0.8)	223 (42)	

**Table 4:** Association between Histopathology of Gastric and HBsAg Positivity in Logistic Regression Analyse

Histopathology of gastric	HBsAg positive/ *H. pylori* negative (n=18)	HBsAg/*H. pylori *negative (n=223)	OR	95% CI	P value
Gastritis	4	5	12.457	3.007-51.614	0.001
Atrophic gastritis	6	0	8.077	-	0.994
Metaplasia	2	9	2.972	0.592-14.932	0.1868
Dysplasia	0	4	0	0	0.999
Cancer	1	6	2.127	0.242-18.704	0.496
Total	13	24	21.56	7.070-65.741	<0.001

Hepatitis B virus (HBV) is a DNA virus that has liver tropism. It can induce hepatic damage and subsequent hepatocellular carcinoma (Ahmadi Vasmehjani et al., 2015). Chronic HBV infection is associated with extra-hepatic cancers (EHCs) such as, pancreatic cancer, non-Hodgkin’s lymphoma and gastric cancer. HBV may infect non-hepatic tissues through the bloodstream and` its antigens can be detected in non-liver organs such as stomach, gastrointestinal tract, pancreas and kidney. It is possible that hepatitis B virus replicates within extra-hepatic tissues and plays an oncogenic role (An et al., 2018).

In recent years, researchers have found an association between hepatitis B surface antigen (HBsAg) and stomach cancer. Chen et al., (2004) were found HBsAg and HBcAg of hebatitis B virus in the gastric mucosa of patients infected with *H. pylori*. They were not observed a significant difference in the expression level of HBV antigen between the *H. pylori* positive and negative patients. It was also reported that the high prevalence of gastric malignancy was linked to liver cirrhosis caused by HBV virus. Wei et al., (2015) were found that HBV was potentially associated with stomach cancer. In a study conducted in Iran, HBV was not associated with gastric malignancy (Ghasemi et al., 2012). 

The majority of studies are conducted in China, where both HBV infection and gastric cancer are endemic. Therefore, it is required that the association between HBV and gastric cancer clear by further studies in the other region of the world. In addition, up to date, the correlation of HBV and precancerous lesions of the stomach has not been clearly elucidated. The present study was aimed to evaluate an association between gastric pathology with hepatitis B virus infection in patients with or without *helicobacter pylori*.

## Materials and Methods

*Study population*

This cross-sectional study performed on 728 patients under endoscopy examination who referred to Yazd Shaheed Sadoughi Hospital between 2017 and 2018. A gastric biopsy and serum sample was taken from each patient. All subjects signed an informed consent form before sample collection. Exclusion criteria were the following: age under 18 years old, the impossibility for perform endoscopy, and difficulty of taking a stomach biopsy. Patient demographic characteristics, including, age, sex, type of digestive disorder, and the history of drug addiction were collected by a questionnaire. The study was approved by the Ethics Committee of Shahid Sadoughi University of Medical Sciences in Yazd (IR.SSU.MEDICINE.REC.1396.263).

*Pathological analysis of gastric specimens*

Tissue samples were fixed in 10% formalin, sliced into smaller sections, and then embedded in paraffin. The paraffin-embedded tissues were cut into five micron segments and stained with hematoxylin-eosin (H and E) and Giemsa for histopathological evaluation of the tissues and detection of *H. pylori* infection. The histological feature of samples and the pathological stage of tissues were estimated by two pathologists according to the American Joint Committee on Cancer criteria.

*Serologic assay for detection of HBV infection*

Hepatitis B surface antigen (HBsAg) in serum was detected by the enzyme-linked immunosorbent assay (ELISA) technique according to the manufacturer’s instruction (Diapro, Italy) with high sensitivity (100%) and specificity (99.5%). 

*Statistical analysis*

Data were analyzed using SPSS 21.0 statistical software (SPSS Inc., Chicago, IL, USA). Comparison of variables between HBsAg positive and negative groups were carried out by Chi-square and t test. Logistic regression analysis was performed to determine the association between histopathology of gastric and HBsAg positivity. A P-value less than 0.05 was considered statistically significant.

## Results

Of 728 patients under study, 370 people (50.8٪) were male and 358 (49.2٪) female. Frequency distribution of population in terms of variables under study is summarized in Table 1. Twenty three patients were addicted and 83 subjects (11.4٪) were HBsAg positive. *H. pylori* infection was detected in 408 patients (56٪) and 69 peoples were also co-infected with *H. pylori*/HBV. 

According to histopathology of gastric, patients were placed in six groups, including, normal, gastritis, atrophic gastritis, metaplasia, dysplasia and cancer. In 259 subjects (35.6٪), histopathology of stomach was normal. Dysplasia had the lowest frequency, while Gastritis was the most common histopathological phenotype (40.1٪) and gastric cancer detected in 35 cases (4.8٪). 

Table 2 shows the association between variables under study and HBsAg detection. A significant association between the mean age (p=0.043), sex (p=0.01), addiction (p=0.031) and HbsAg positivity was observed. In addition, *H. pylori* infection detected in patients with HbsAg positive significantly more than those were negative for HbsAg (p=0.029, Table 2).

Histopathology findings between HBsAg positive and HBsAg negative patients are compared and summarized in Table 3. None of HBV/*H. pylori* co-infected patients did not have normal gastric tissue and gastritis was the most frequently histological finding in them. The majority of HBsAg positive/*H. pylori* negative patients had atrophic gastritis. There is a significant difference in histopathology of gastric tissue between HBsAg positive patients with and without *H. pylori* infection (p<0.0001, Table 3). Fifty five HBsAg negative/ *H. pylori* positive patients had normal gastric tissue. *H. pylori* infection was significantly associated with gastric cancer either in patients with or without HBV infection. Precancerous conditions and cases of gastric cancer in *H. pylori* positive patients were founded to be more than patients without *H. pylori* infection. Logistic regression analyses showed that the presence of HBsAg is associated with histopathology of gastric (Table 4, OR=21.56, 95℅CI=7.070-65.741, p<0.001). The HBsAg was as a risk factor for gastritis (OR=12.457, 95℅CI= 3.007-51.614, P=0.001) but no cancer (OR=2.127, 95℅CI=0.242-18.704, P=0.496). 

## Discussion

Recent studies have reported hepatitis B virus infection associated with gastric mucosal lesions and stomach carcinoma. Results of the present study showed that HBV infection is significantly linked to increasing risk of developing precancerous conditions but no gastric cancer. The role of Helicobacter in the development of gastric cancer was more intense than the hepatitis B virus. The results of the present study introduce HBV as a possible risk factor or cofactor for gastric carcinoma only in coinfection with *H. pylori.*

Many previous studies have obtained a positive strong relationship between *Helicobacter pylori* infection and gastric lesions, so that the World Health Organization considered this bacterium as a class 1 carcinogen for gastric cancer (Wang et al., 2016). The role of HBV infection in the development of hepatocellular carcinoma is confirmed by previous studies, but limited reports have shown an association between this virus with other malignancies such as renal cancer and gastric cancer (Ghasemi et al., 2012). Malignancy is a multifactor disease and HBV infection is a risk factor for liver cirrhosis and hepatocellular carcinoma (Hosseini et al., 2011). Several studies introduced liver cirrhosis as a risk factor for gastric cancer (Zullo et al., 2003; Kirchner et al., 2011). Therefore, HBV infection and gastric cancer may be linked by liver cirrhosis. On the other hand, HBsAg is detected in gastric epithelial cells (Chen et al., 2004; Wei et al., 2015), thus it is possible that HBV directly increases the risk of gastric cancer. Stomach is adjacent to liver and may be infected by HBV. In the current study, HBsAg was detected in 11.4℅ of patients suffering from gastric cancer. In a study conducted in China, the existence of HBsAg in ten common extra-hepatic cancers was evaluated. About 14℅ of patients with gastric cancer were HBsAg positive (Lu et al., 2018). In Republic of Korea, HBsAg was positive in 4.7℅ of men and 3.4℅ of women with stomach cancer (An et al., 2018).

In the present study, cases of precancerous and gastric cancer in HBV/*H.pylori* co-infected patients were significantly more than *H. pylori* positive/HBV negative patients (Table 3, p<0.0001). This finding shows that HBV infection may contribute in progression of gastric cancer. Unlike the findings of the current study, some previous studies have not founded a link between *H. pylori* and HBV infection in gastric lesions. Chen et al., (2004) were detected HBsAg and HBcAg in the gastric mucosa infected with *H. pylori*, but they were not obtained a significant difference in expression of HBsAg between the *H. pylori*-positive and -negative gastric tissues in HBV-infected patients.

In the current study, only one patients with gastric cancer was HBsAg positive/*H. pylori* negative; therefore, the role of hepatitis B infection alone and directly in the development of stomach cancer is not strong (Table 4, OR=2.127, 95℅CI=0.242-18.704, P=0.496). Unlike this finding, Wei et al., (2015) were reported a positive association between HBV infection and gastric cancer in a case-control study. They did not evaluate the subjects for *Helicobacter pylori* infection, thus may be infection with *H. pylori* has been effective in them results. In a study conducted in north of Iran, HBV was not associated with gastric malignancy because viral DNA was undetectable in all one hundred samples (Ghasemi et al., 2012). We were not performed the PCR for detecting viral genome and it was a limitation for this study. Based on literature review, the HBV genome is detected in less than 5℅ of gastric cancer tissues (Chen et al., 2015); therefore, further research with large sample size is needed to confirm the relation between this virus and gastric carcinoma. 

In conclusion, this study showed that HBV infection alone is associated with some precancerous lesions but is not correlated with gastric cancer. HBV can increase development of premalignant conditions and carcinoma significantly in patients infected with *H. pylori*.
